# Toward Generalized Emotion Recognition in VR by Bridging Natural and Acted Facial Expressions

**DOI:** 10.3390/s26030845

**Published:** 2026-01-28

**Authors:** Rahat Rizvi Rahman, Hee Yun Choi, Joonghyo Lim, Go Eun Lee, Seungmoo Lee, Chungyean Cho, Kostadin Damevski

**Affiliations:** 1 Department of Computer Science, College of Engineering, Virginia Commonwealth University, Richmond, VA 23220, USA; kdamevski@vcu.edu; 2 School of Film, TV & Multimedia, Korea National University of Arts, Seoul 02789, Republic of Korea

**Keywords:** human emotion recognition, virtual reality, facial action coding system, domain adaptive neural networks, mixture-of-experts

## Abstract

Recognizing emotions accurately in virtual reality (VR) enables adaptive and personalized experiences across gaming, therapy, and other domains. However, most existing facial emotion recognition models rely on acted expressions collected under controlled settings, which differ substantially from the spontaneous and subtle emotions that arise during real VR experiences. To address this challenge, the objective of this study is to develop and evaluate generalizable emotion recognition models that jointly learn from both acted and natural facial expressions in virtual reality. We integrate two complementary datasets collected using the Meta Quest Pro headset, one capturing natural emotional reactions and another containing acted expressions. We evaluate multiple model architectures, including convolutional and domain-adversarial networks, and a mixture-of-experts model that separates natural and acted expressions. Our experiments show that models trained jointly on acted and natural data achieve stronger cross-domain generalization. In particular, the domain-adversarial and mixture-of-experts configurations yield the highest accuracy on natural and mixed-emotion evaluations. Analysis of facial action units (AUs) reveals that natural and acted emotions rely on partially distinct AU patterns, while generalizable models learn a shared representation that integrates salient AUs from both domains. These findings demonstrate that bridging acted and natural expression domains can enable more accurate and robust VR emotion recognition systems.

## 1. Introduction

Emotion recognition enables VR systems to dynamically adapt to users’ affective states (e.g., happiness, sadness, fear), thereby creating more immersive and responsive virtual environments [1]. By interpreting the emotional signals of the person wearing the headset, VR applications can adjust feedback, narrative flow, or difficulty levels in real time [2,3,4,5]. For example, a monster-inspired VR game can modify its pacing and audiovisual intensity to sustain engagement based on the player’s fear response [4]. While emotion can be inferred from diverse modalities such as speech and physiological activity, facial expression recognition (FER) remains one of the most direct and informative signals of a user’s affective state. FER is typically based on a compact representation of muscle activity using the facial action coding system (FACS) [6,7]. FACS describes facial muscle movements as discrete action units (AUs), such as AU6 (Cheek Raiser) and AU12 (Lip Corner Puller), which together form the basis for interpreting complex emotional expressions.

Recent advances in headset-integrated sensing have enabled FER to move beyond external camera setups. Devices such as the Meta Quest Pro and Apple Vision Pro use inward-facing infrared cameras to capture detailed facial motion data, even under occlusion [8,9,10]. This allows emotion recognition directly within immersive experiences, supporting applications in social interaction, gaming, training, and therapy [1,11]. However, most current FER systems in VR rely on acted emotional expressions, which are collected under controlled conditions and tend to exaggerate affective cues [12,13]. Although acted expressions are easier to collect and annotate, they differ substantially from how emotions are naturally expressed during real-world interactions. Natural expressions occur spontaneously, are less intense, and exhibit greater temporal variability [14,15].

Our prior work showed that natural and acted emotional expressions in VR differ in both spatial and temporal dynamics [16]. Natural emotions engage different facial AUs, vary more over time, and are generally more subtle. As a result, FER models trained on acted data degrade when applied to spontaneous emotional behavior [14,17].

Nonetheless, models trained on acted data remain valuable, as people sometimes express emotions in exaggerated ways, especially in social, gaming, or performative settings. These models capture the clear, high-intensity patterns that occur when emotions are strongly displayed. However, they lack sensitivity to the subtle, spontaneous expressions typical of everyday interactions. Models trained jointly on acted and natural data can bridge this gap by learning a continuous representation of expressive variability that more accurately reflects the full range of real-world emotional behavior.

To motivate the need for cross-domain, generalizable modeling, we emphasize three key considerations:

1. *Coverage of the expressive spectrum.* Acted and natural emotions occupy different regions of affective space. Training on both domains exposes the model to the full continuum of expression intensity, improving robustness to subtle or blended affective cues that occur naturally [14,15].
2. *Consistency and user trust.* Emotionally adaptive VR systems that misinterpret or ignore familiar acted-like expressions appear inconsistent or unintelligent to users. Conversely, systems that respond only to exaggerated displays fail to recognize genuine, low-intensity emotion. Models that generalize across both expression types enable predictable affective feedback, increasing user trust and the perceived accuracy of emotion-aware interactions [1,11].
3. *On-device emotion recognition.* Our goal is to perform emotion recognition directly on the VR headset to ensure real-time responsiveness. Executing inference locally eliminates network latency, maintaining immediate and stable emotional feedback. This real-time capability benefits from a unified, generalizable model that is practical for interactive VR scenarios, where even a minimal delay can disrupt immersion and diminish the sense of presence.

We investigate two research questions in this work: (1) Can a model trained jointly on natural and acted emotion data recognize emotions more effectively across both domains than models trained on a single domain? (2) How do the facial action units that drive predictions in the generalized model differ across natural and acted emotions? The first question addresses the feasibility of developing emotion recognition systems that generalize beyond a single expression type. The second question examines the underlying facial behaviors captured by the generalized model, providing insight into how natural and acted emotions manifest differently in VR.

Our findings indicate that training on both natural and acted data improves emotion recognition performance on natural expressions and combined test sets, compared to single-domain models. The generalized models, particularly those using domain-adversarial neural networks and mixture-of-experts architectures, achieve the best overall results. Additionally, analysis of feature importance reveals distinct facial action unit (AU) patterns associated with natural versus acted emotions. These patterns highlight differences in facial muscle engagement across domains. This work advances the development of robust, real-time emotion recognition systems for VR that can adapt to the full spectrum of human emotional expression.

The main contributions of this work can be summarized as follows:

- We investigate emotion recognition in virtual reality by jointly modeling natural and acted facial expressions using facial action unit data captured from headset-integrated sensors.
- We systematically compare convolutional, domain-adversarial, and mixture-of-experts architectures to evaluate their ability to generalize across expression domains.
- We show that cross-domain training improves robustness and generalization to natural and mixed-expression test settings compared to single-domain models.
- We provide an interpretable analysis of facial action units using SHAP to reveal how generalized models integrate features from both natural and acted expressions.

## 2. Methodology

In this section, we describe the datasets, preprocessing steps, model architectures, evaluation protocols, and implementation details used in our study to develop generalized emotion recognition models for VR applications.

### 2.1. Data Sources and Collection

Our study integrates two complementary datasets that together capture the continuum of human emotional expression in virtual reality: (1) *VR Natural–Acted Emotion Dataset* from Tare et al. [16], and (2) *EmojiHeroVR Dataset* from Ortmann et al. [9]. Both datasets were recorded using the Meta Quest Pro headset, which provides inward-facing infrared cameras capable of tracking facial muscle activity through the facial action coding system (FACS).

The *VR Natural–Acted Emotion Dataset* contains 34 participants (ages 20–60) recorded under two conditions. In the natural condition, participants viewed short emotionally evocative videos designed to elicit one of seven target emotions: happiness, sadness, anger, fear, disgust, surprise, and neutral. While participants watched these videos, their AU activations were recorded at 10 Hz. After each clip, participants reported the type, intensity, and frequency of emotions experienced using a structured questionnaire. In the acted condition, the same participants intentionally reenacted each target emotion for approximately one minute. The resulting dataset comprises synchronized AU time series and self-reported emotion annotations, enabling analysis of both spontaneous and deliberate facial movements within the same individuals.

The *EmojiHeroVR Dataset* provides complementary acted data where 37 participants (ages 19–50) played an affective VR game, called EmojiHeroVR, where they reenacted emotions corresponding to on-screen emoji prompts representing the same seven basic emotions. During gameplay, the Meta Quest Pro headset captured 63 AU activation values per frame via its Face Tracking API. After collection, three independent annotators labeled each reenacted frame sequence, achieving substantial inter-rater agreement (Fleiss’ Kappa = 0.68). The final dataset includes 1727 synchronized AU activation sequences recorded at 30 Hz.

Together, these two datasets provide complementary coverage of natural and acted emotions in VR: *VR Natural–Acted Emotion Dataset* contributes natural, self-reported emotional behavior, while the *EmojiHeroVR Dataset* supplies large-scale acted expressions. While *VR Natural–Acted Emotion Dataset* also includes acted expressions; the volume of data is comparatively limited. Combining the two datasets enables cross-domain modeling of facial emotion recognition that spans the spectrum of affective behavior within immersive virtual environments.

### 2.2. Data Preprocessing

The goal of preprocessing was to enable joint analysis across the two datasets by constructing a unified representation of facial activation dynamics. Since both the *VR Natural–Acted Emotion Dataset* and *EmojiHeroVR Dataset* were collected using the Meta Quest Pro headset and relied on the same Face Tracking API, they shared a consistent set of 63 AU activation values ranging from 0 to 1. This common structure allowed direct integration once sampling and labeling differences were reconciled.

The primary discrepancy between the two datasets was their temporal resolution. The *EmojiHeroVR Dataset* sequences were recorded at 30 Hz, while the AU data in *VR Natural–Acted Emotion Dataset* were sampled at 10 Hz. To achieve temporal consistency, we downsampled the *EmojiHeroVR Dataset* to 10 Hz by averaging non-overlapping windows of three consecutive frames for each AU channel.

### 2.3. Model Architectures

We evaluate four model architectures across three dataset configurations to examine their ability to generalize across both natural and acted emotional expressions in VR. The models include two standard approaches and two domain-aware extensions: (1) a convolutional network using 1-D convolutions (Conv1D), (2) a temporal convolutional network (TCN) with dilated causal convolutions, (3) a domain-adversarial neural network with a Conv1D backbone (DANN-Conv1D), and (4) a domain-adversarial neural network with a TCN backbone (DANN-TCN). To further assess domain-specific specialization, we also evaluate a mixture-of-experts (MoE) architecture designed to combine natural and acted emotional dynamics. We describe each of these models, in turn, below:

#### 2.3.1. One-Dimensional Convolutional Neural Network (Conv1D)

The Conv1D architecture consists of two stacked causal 1-D convolutional layers with 128 filters, kernel size 4, and ReLU activations. The first convolution extracts short-range temporal patterns, while the second layer employs a dilation rate of 2 to capture longer-range dependencies efficiently. A dropout layer mitigates overfitting, followed by a global average pooling layer to aggregate temporal features into a compact embedding. The final dense output layer applies a softmax activation to produce emotion class probabilities. Conv1D is a popular approach for local temporal representation learning.

#### 2.3.2. Temporal Convolutional Network (TCN)

The TCN architecture replaces standard convolutions with dilated causal convolutions to model extended temporal dependencies while preserving sequence order. It uses 128 filters and a kernel size of 4, with dilation factors generated via a custom schedule to ensure full temporal coverage. The extracted temporal features pass through two dense layers with 256 and 128 hidden units, ending with a softmax classifier. The TCN’s exponential dilation pattern allows efficient context aggregation, enabling robust modeling of facial AU transitions over longer time spans.

#### 2.3.3. Domain-Adversarial Neural Network (DANN)

To address the domain shift between natural and acted expressions, we implemented the DANN-Conv1D and DANN-TCN models. Both follow a two-head design: an emotion classifier head and a domain classifier head, connected through a gradient reversal layer (GRL) that inverts gradients from the domain branch to enforce domain-invariant representations.

- DANN-Conv1D uses a shared feature extractor composed of two Conv1D layers (128 filters, kernel size 3) with batch normalization and global average pooling. The emotion classifier head applies a dense layer (128 units) and then a softmax layer, while the domain classifier mirrors this design after the GRL. Class-balanced weighted cross-entropy losses are applied independently to both heads.
- DANN-TCN replaces the convolutional backbone with three residual TCN blocks with dilation rates 1, 2, and 4. Each block includes paired causal convolutions, batch normalization, and skip connections for stable deep temporal modeling. The pooled latent features are fed into dual classifier heads identical to the Conv1D version.

#### 2.3.4. Mixture of Experts (MoE)

The MoE model captures the differences between natural and acted expressions using three parallel expert networks: a *Natural Expert*, an *Acted Expert*, and a *Shared Expert*. Each expert is built with three stacked temporal convolutional network (TCN) blocks (128 filters, kernel size 3, dilation rates 1, 2, and 4), followed by global average pooling to produce a compact feature vector. A softmax-based gating network selects the two most relevant experts for each input, with temperature annealing over training to sharpen expert selection. Finally, the weighted combination of expert outputs is passed through a dense layers with 256 units and a softmax classifier to predict the emotion label.

### 2.4. Evaluation Protocol

To enable robust evaluation across models and domains, we adopted subject-exclusive, class-balanced data splits and standardized performance metrics. The evaluation was conducted independently for the natural, acted, and combined datasets to assess both within-domain and cross-domain generalization.

For the *VR Natural–Acted Emotion Dataset*, we partitioned the data into training, validation, and test sets using a 70/10/20 split at the subject level. In other words, if participant A’s recordings were used for training, none of their data would be used for validation or testing. This approach ensures that the model is evaluated on unseen individuals, preventing it from memorizing personal patterns rather than learning generalizable emotional cues. We further stratified the data to maintain class balance across the seven target emotions. For the *EmojiHeroVR Dataset*, we used the official train, validation, and test partitions, which similarly maintain subject separation and balanced class distributions.

Following our prior work [16], we used sliding windows of 5 frames (0.5 s at 10 Hz) with a stride of 1 frame. Each window was treated as an independent training or evaluation instance and labeled according to the dominant emotion class of the original sequence. This approach enables the models to use some context, which enhances the stability of predictions to frame-level variability.

After preprocessing and sliding-window generation, the resulting class-wise sample distribution remained imbalanced across emotions and datasets. In the natural dataset, in *VR Natural–Acted Emotion Dataset*, training samples per emotion ranged from 313 (Surprise) to 1422 (Happiness), with corresponding test samples ranging from 51 (Neutral) to 488 (Disgust). For acted data, the *VR Natural–Acted Emotion Dataset* contributed between 189 and 253 training samples per emotion, while the *EmojiHeroVR Dataset* provided substantially larger volumes, ranging from 546 to 2228 training samples per class. A similar imbalance was observed in the test splits, particularly for acted emotions.

We evaluated three dataset configurations: (1) natural-only (training on natural data), (2) acted-only (training on acted data from both *VR Natural–Acted Emotion Dataset* and *EmojiHeroVR Dataset*), and (3) generalized (training on the combined natural and acted training sets). Testing was performed separately on the natural, acted, and combined test partitions to measure both domain-specific and cross-domain performance. All models were evaluated using weighted accuracy, precision, recall, and F1-score to account for class imbalance.

### 2.5. Implementation Details

All models were implemented in TensorFlow Keras using standard Conv1D, Dense, and TCN layers. For the TCN, we employed causal dilated temporal convolutions with exponentially increasing dilation factors (powers of two), chosen such that the receptive field covers the full input sequence length. The DANN models included a custom gradient reversal layer, and the MoE model used a Top-2 gating mechanism with temperature annealing for expert selection. Training used the SGD optimizer (learning rate 0.01, momentum 0.95), except DANN and MoE, which used a learning rate of 0.001. For all the models we used sparse categorical cross-entropy loss. Each model was trained separately on the natural, acted, and generalized training datasets for 30 epochs with a batch size of 10. Accuracy served as the main validation metric.

## 3. RQ1: Can a Model Trained Jointly on Natural and Acted Emotion Data Recognize Emotions More Effectively Across Both Domains than Models Trained on a Single Domain?

Table 1 presents the performance comparison of all evaluated models across the natural, acted, and combined test sets. Models trained exclusively on a single domain perform best within that domain but exhibit sharp degradation when evaluated on the other domain, consistent with prior findings [16]. For example, the acted Conv1D model achieves an accuracy of 0.49 on the acted test set but falls to 0.21 on the natural test set, indicating a clear domain gap between acted and natural expressions. Similarly, the natural Conv1D model performs adequately on the natural data (Acc = 0.28) but drops to 0.23 on acted data. This trend holds across model architectures, with consistently higher scores on acted test sets, reflecting the more exaggerated and easily distinguishable nature of acted emotions.

The generalized models, trained jointly on both natural and acted datasets, exhibit improved balance and robustness across domains. The generalized Conv1D model attains F1-scores of 0.30 and 0.43 on the natural and acted test sets, respectively, outperforming the domain-specific Conv1D models in cross-domain evaluation. On the combined test set, it reaches F1 = 0.39, outperforming the natural-only configuration (F1 = 0.24) and approaching the acted model (F1 = 0.41). A similar trend appears in the TCN models, where the generalized version (F1 = 0.38) exceeds the natural TCN (F1 = 0.23) on the combined set, indicating that exposure to heterogeneous emotional data enables stronger domain-general feature learning.

The domain-adversarial neural network (DANN) models further enhance cross-domain consistency, yielding noticeable gains relative to the generalized baselines. The DANN-TCN model, for instance, reaches F1 = 0.43 on the combined test set, surpassing the generalized TCN (F1 = 0.38). These improvements suggest that adversarial domain alignment helps reduce the representation gap between acted and natural expressions, though it does not entirely eliminate it.

The mixture-of-experts (MoE) model delivers the strongest overall results. It achieves an accuracy of 0.34 on the natural test set and 0.48 on the acted test set, leading to 0.44 accuracy and F1 = 0.43 on the combined evaluation. This result demonstrates that explicitly modeling domain specialization through multiple expert pathways, combined with a shared gating mechanism, captures both subtle and exaggerated emotional patterns more effectively.

For RQ1, these results confirm that models trained jointly on both natural and acted emotion data recognize emotions more effectively across domains than those trained on a single domain. The consistent improvement observed in generalized, DANN, and MoE configurations highlights the advantage of learning from mixed-domain data. Although acted models maintain a narrow advantage within their own domain, for natural and combined emotion recognition, the DANN and MoE models achieve the strongest results.

## 4. RQ2: How Do the Facial Action Units That Drive Predictions in the Generalized Model Differ Across Natural and Acted Emotions?

We employed SHAP (Shapley additive explanations) analysis to identify the most important facial action units (AUs) for emotion prediction across three models: one trained exclusively on natural expressions (Natural TCN+Dense), one on acted expressions (Acted TCN+Dense), and a combined model trained on both (Generalized TCN+Dense). SHAP values quantify the contribution of each feature to the prediction for specific instances. For our SHAP analysis, we evaluated each model on its corresponding data distribution: the natural model on 100 random natural samples, the acted model on 100 random acted samples, and the combined model on 100 random mixed samples. This approach allows us to understand how each model characterizes the AUs it was specifically designed to recognize.

In the natural model, the most influential AUs are primarily located around the eyes and cheeks, including LidTightenerL, CheekRaiserL, and UpperLipRaiserL, along with balanced contributions from their right-side counterparts. These AUs correspond to fine-grained muscle activations such as gentle squinting, cheek elevation, and minor lip movement, all of which are typical of spontaneous and subtle emotional expressions. The dominance of these upper-face features indicates that natural emotional reactions in VR tend to manifest through subtle facial muscle shifts rather than broad, high-intensity gestures.

On the other hand, in the acted model, the dominant AUs represent high-intensity facial movements, including JawDrop, UpperLidRaiserR, and LidTightenerL, which reflect deliberate and overt expressions. These AUs represent movements like opening the mouth widely, lifting the eyelids, and tightening the eyelids to amplify visibility of emotion. Compared to the natural model, these AUs engage lower-face regions more strongly and exhibit higher amplitude and symmetry, creating clear, prototypical emotion patterns that are easy for observers (and models) to recognize.

**Figure 1:**
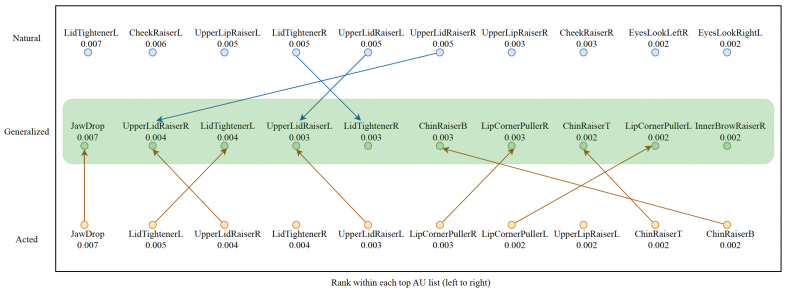
SHAP-based feature importance showing the top 10 facial action units (AUs) for TCN+Dense models trained on natural, acted, and generalized datasets, ordered from highest to lowest importance from left to right. Arrows indicate action units shared between the generalized model and the natural or acted models.

In the generalized model, the top AUs combine characteristics of both natural and acted domains, featuring both JawDrop (from acted expressions) and LidTightenerL or UpperLidRaiserR (from natural expressions). This overlap, illustrated by arrows in the Figure 1, demonstrates that the model learns to integrate subtle eye-region cues with the more pronounced mouth and jaw activations, achieving a hybrid expressive representation.

For RQ2, we can conclude that the facial AUs driving emotion prediction differ systematically between natural and acted emotions, confirming that these two domains are distinct yet complementary. The generalized model bridges these by simultaneously emphasizing AUs from both regions, demonstrating that exposure to both domains allows it to internalize a shared, domain-invariant representation of emotional behavior. Thus, RQ2 is answered by showing that generalizable emotion recognition in VR depends on integrating the fine-grained, spontaneous cues of natural emotion with the intense, well-defined cues of acted emotion.

## 5. Discussion

To contextualize our results with respect to prior work, we compare our performance against baselines established in the source datasets. In the acted domain, Ortmann et al. [9] reported classification accuracies of up to 69.84% using headset-integrated facial muscle sensors. While our best acted-only performance is lower (Acc = 0.50), this difference is largely attributable to methodological choices designed to support cross-domain evaluation, specifically downsampling the acted data of *EmojiHeroVR Dataset* from 30 Hz to 10 Hz to match our dataset. This downsampling removes fine-grained temporal cues that are informative for acted expressions, resulting in a more challenging but consistent evaluation setting across domains.

Notably, Tare et al. [16] reported an F1-score of 0.22 when models trained on acted data were applied to natural expressions. In contrast, our generalized mixture-of-experts model achieves an F1-score of 0.34 on the natural test set under the same AU intensity threshold. These results indicate that the proposed multi-expert framework more effectively bridges the domain gap between acted and natural emotional expressions in VR.

## 6. Threats to Validity

In this section, we discuss potential threats to the validity of our study, organized into construct validity, internal validity, and external validity.

Construct validity concerns whether the operationalization of our constructs accurately reflects the theoretical concepts of interest. One potential threat arises from our reliance on datasets collected through different elicitation procedures for natural and acted emotions. Although both datasets were obtained using the Meta Quest Pro headset and share consistent AU tracking interfaces, differences in task design and emotional prompting may introduce confounding factors unrelated to the naturalness of expression itself. To mitigate this threat, we aligned sampling frequencies, harmonized AU features, and employed consistent preprocessing strategies across datasets. Another potential threat concerns the limited reliability of self-reported emotion annotations in the *VR Natural–Acted Emotion Dataset*. Participants may have experienced blended or ambiguous affective states, which could reduce the accuracy of ground-truth labels. To address this, we used high thresholds of participants’ self-reported emotions (intensity of 5 or above on a 10-point Likert scale) to select the data. A further validity concern relates to the SHAP analysis, since each model is evaluated on its corresponding data distribution rather than on identical samples. While this setup reflects feature importance within each model’s target domain, the resulting differences capture both inherent expression characteristics and model-specific representational biases. Finally, the models’ performance may also be influenced by hyperparameter settings or architecture-specific inductive biases.

Internal validity pertains to whether the observed outcomes can be attributed to our modeling approach rather than uncontrolled variables. A possible threat is participant variability, including individual differences in expressiveness, facial morphology, and cultural display norms, which may confound the relationship between domain (natural versus acted) and model performance. We reduced this threat by using subject-exclusive splits to ensure that each participant’s data appeared in only one partition. Another source of internal threat is model overfitting, especially when training on limited natural data. To counteract this, we used dropout regularization, balanced cross-entropy losses, and early stopping. Additionally, differences in recording conditions or session lighting could have affected AU detection fidelity, though all data were captured under similar headset-based infrared setups, reducing environmental variance. Lastly, while the DANN and MoE models were designed to separate domain-specific and domain-invariant patterns, there remains the possibility that latent correlations between domains persist, influencing observed generalization improvements.

External validity refers to the extent to which our findings can be generalized to other settings, devices, or user populations. One limitation is that both datasets were collected using the Meta Quest Pro headset. Although its AU tracking capabilities are representative of modern VR systems, models trained on this data may not directly transfer to headsets with different camera configurations or tracking algorithms, such as the Apple Vision Pro. Another potential threat arises from the cultural and demographic composition of participants, which may not capture the diversity of global emotional expressiveness. The generalization of our results to different populations should therefore be approached with caution. Furthermore, our study focused on seven basic emotions and did not account for complex or compound affective states that could occur in interactive VR environments. Finally, we evaluated model generalization primarily across two controlled datasets rather than in-the-wild user experiences.

## 7. Related Work

In virtual reality, emotion recognition faces challenges due to the occlusion of facial regions by head-mounted displays [13,18]. Conventional camera-based face emotion recognition (FER) systems lose access to key features such as eye and brow movements once users wear VR headsets. Ortmann et al. [9] demonstrated that occlusion leads to a steep drop in FER accuracy from above 90% with full facial visibility to below 70% when the upper face is covered. To mitigate this issue, some modern VR headsets integrate inward-facing infrared cameras that capture facial muscle activity directly from within the device [8]. These sensors estimate activations of the facial action units (AUs) defined by the facial action coding system (FACS) [6], providing a structured and interpretable description of facial movements. The FACS framework [19,20] has been widely adopted for emotion analysis and has become the foundation for recent VR-based FER systems. Devices such as the Meta Quest Pro and Apple Vision Pro support AU estimation in real time, enabling on-device emotion recognition and affect-driven interaction [1,11]. Accessory systems like the HTC VIVE Facial Tracker and Emteq Pro [8] extend this capability to legacy headsets, though typically with partial coverage of the face. Ortmann et al. [9,10] leveraged these inward-facing sensors to create the EmojiHeroVR dataset, demonstrating that FACS-aligned AU data can support reliable classification of acted emotions even under partial occlusion.

While most VR FER studies rely on acted emotions, several works have examined the differences between natural and acted expressions. Ekman and Friesen [6] established that genuine smiles involve the activation of AU6 (*Cheek Raiser*) and AU7 (*Lid Tightener*), features typically absent in acted expressions. Mavadati et al. [17] extended this distinction using the DISFA+ dataset, comparing acted and natural facial expressions in naturalistic settings and confirming that acted emotions exhibit stronger, more temporally consistent AU activations. Namba et al. [14] similarly reported strong differences between spontaneous and posed facial behavior. More recently, Tare et al. [16] studied this phenomenon in VR using inward-facing sensors on the Meta Quest Pro headset. Their findings showed that natural and acted emotions differ in both the magnitude and temporal evolution of AU activations, and that models trained on acted VR data degrade substantially when applied to natural emotions.

This paper builds directly on these findings. Unlike prior studies that focus exclusively on acted or natural emotions, we integrate both domains to train unified models capable of recognizing the full spectrum of facial expressivity in VR. By combining two complementary VR datasets, one natural and one acted, and leveraging domain-adaptive and mixture-of-experts architectures, we aim to bridge the expressive gap identified in earlier research.

## 8. Conclusions and Future Work

This study examined how integrating acted and natural emotional expressions can improve the generalization of facial emotion recognition models in VR. We combined two complementary datasets collected with the Meta Quest Pro headset and evaluated several individual and cross-domain (generalized) model architectures. The results show that models trained cross-domain outperform single-domain models, with the DANN and mixture-of-experts configurations achieving the most balanced accuracy across all three test sets (natural, acted, and combined). A feature importance analysis using SHAP revealed that generalizable models learn representations combining subtle AUs from natural expressions and pronounced AUs from acted ones, resulting in more robust recognition across expression types.

The findings of this study demonstrate the importance of cross-domain modeling for robust emotion recognition under realistic VR conditions. The outcomes of this work advance the development of adaptive VR systems that can respond to users’ genuine emotional states in real time. In practical VR systems, emotion recognition is typically used as a temporally aggregated probabilistic control signal rather than a frame-level decision, allowing moderate per-window accuracy to remain effective while enabling real-time, on-device deployment using headset-integrated facial sensing. Generalizable emotion models enhance the realism of interaction and support a wider range of affective computing applications, including immersive therapy, training, and entertainment. By capturing both authentic and performative cues, these models improve the system’s ability to interpret diverse affective behaviors and build user trust during interaction.

Building on the contributions of this work, future research will extend this framework through multimodal affect recognition that incorporates vocal and physiological signals. We plan to refine the mixture-of-experts design for real-time, on-device inference and explore continuous emotion modeling in different VR applications. Expanding data collection to diverse users, contexts, and devices will further test the scalability of this approach. The ultimate goal is to enable emotionally intelligent VR systems that perceive, learn, and adapt to authentic human affect across a full spectrum of expressive behavior. 

## Figures and Tables

**Table 1:** Performance comparison of Conv1D, TCN, domain-adversarial (DANN), and mixture-of-experts (MoE) models evaluated on natural, acted, and combined test sets. Metrics include accuracy (Acc) and weighted precision (Prec), weighted recall (Rec), and weighted F1-score (F1). The bold values indicate the highest performance within each column.

Model	Natural Test Set				Acted Test Set				Combined Test Set			
	Acc	Prec	Rec	F1	Acc	Prec	Rec	F1	Acc	Prec	Rec	F1
Conv1D Models												
Natural Conv1D	0.28	0.32	0.28	0.28	0.23	0.24	0.23	0.22	0.24	0.26	0.24	0.24
Acted Conv1D	0.21	0.31	0.21	0.22	**0.49**	**0.50**	**0.49**	**0.49**	0.41	0.43	0.41	0.41
Generalized Conv1D	0.28	0.38	0.28	0.30	0.44	0.45	0.44	0.43	0.39	0.40	0.39	0.39
DANN-Conv1D	**0.34**	**0.45**	**0.34**	0.34	0.47	0.48	0.47	0.45	0.43	**0.45**	0.43	0.42
TCN Models												
Natural TCN	0.31	0.40	0.31	0.34	0.21	0.22	0.21	0.20	0.24	0.25	0.24	0.23
Acted TCN	0.19	0.26	0.19	0.19	0.47	0.47	0.47	0.46	0.39	0.40	0.39	0.39
Generalized TCN	0.28	0.33	0.28	0.29	0.42	0.43	0.42	0.42	0.38	0.38	0.38	0.38
DANN-TCN	**0.34**	0.43	**0.34**	**0.35**	0.48	0.48	0.48	0.46	**0.44**	0.44	**0.44**	**0.43**
Mixture-of-Experts Models												
MoE (3 Experts, Top-2)	**0.34**	**0.45**	**0.34**	0.34	0.48	0.46	0.48	0.46	**0.44**	0.44	**0.44**	**0.43**

## Data Availability

The data presented in this study are available on request from the corresponding author due to privacy and ethical restrictions.

## References

[1] Visconti A., Calandra D., Giorgione F., Lamberti F. (2025). Enhancing Social Experiences in Immersive Virtual Reality with Artificial Facial Mimicry. IEEE Trans. Vis. Comput. Graph..

[2] Dehghani F., Zaman L. (2023). Facial emotion recognition in VR games. Proceedings of the 2023 IEEE Conference on Games (CoG).

[3] Subramanian B., Kim J., Maray M., Paul A. (2022). Digital Twin Model: A Real-Time Emotion Recognition System for Personalized Healthcare. IEEE Access.

[4] Ribeiro I.M., Padilha R., Vitorino G., Batista M., Oliveira Y., Almeida J.V., Costa W., Araújo C., Teichrieb V. (2024). Eyes of Fear: Leveraging Emotion Recognition for Virtual Reality Experience. Proceedings of the 26th Symposium on Virtual and Augmented Reality (SVR ’24).

[5] Yu Y., He B., Yu G., Zhong F. (2023). Research on Fear Mental Resilience Training Based on Virtual Reality and Dynamic Decision Fusion. Proceedings of the 2023 International Conference on Pattern Recognition, Machine Vision and Intelligent Algorithms (PRMVIA).

[6] Ekman P., Friesen W.V. (1978). Facial Action Coding System: Investigator’s Guide.

[7] Hess U., Adams R.B., Kleck R.E. (2004). Facial appearance, gender, and emotion expression. Emotion.

[8] Wen L., Zhou J., Huang W., Chen F. (2022). A Survey of Facial Capture for Virtual Reality. IEEE Access.

[9] Ortmann T., Wang Q., Putzar L. (2024). EmojiHeroVR: A Study on Facial Expression Recognition under Partial Occlusion from Head-Mounted Displays. arXiv.

[10] Ortmann T., Wang Q., Putzar L. (2025). Unimodal and Multimodal Static Facial Expression Recognition for Virtual Reality Users with EmoHeVRDB. Proceedings of the 2025 IEEE International Conference on Artificial Intelligence and eXtended and Virtual Reality (AIxVR).

[11] Pan Y., Zhang R., Cheng S., Tan S., Ding Y., Mitchell K., Yang X. (2023). Emotional Voice Puppetry. IEEE Trans. Vis. Comput. Graph..

[12] Jing A., Teo T., McDade J., Zhang C., Wang Y., Mitrofan A., Thareja R., Shin H., Lee Y., Gil Y.H. (2024). Superpowering Emotion Through Multimodal Cues in Collaborative VR. Proceedings of the 2024 IEEE International Symposium on Mixed and Augmented Reality (ISMAR).

[13] Zhang Z., Fort J.M., Giménez Mateu L. (2023). Facial expression recognition in virtual reality environments: Challenges and opportunities. Front. Psychol..

[14] Namba S., Makihara S., Kabir R.S., Miyatani M., Nakao T. (2017). Spontaneous facial expressions are different from posed facial expressions: Morphological properties and dynamic sequences. Curr. Psychol..

[15] Lucey P., Cohn J.F., Kanade T., Saragih J., Ambadar Z., Matthews I. (2010). The extended Cohn-Kanade dataset (ck+): A complete dataset for action unit and emotion-specified expression. Proceedings of the 2010 IEEE Computer Society Conference on Computer Vision and Pattern Recognition-Workshops.

[16] Tare T., Rahman R.R., Choi H.Y., Lim J., Lee G.E., Lee S., Cho C., Damevski K. (2025). Detecting Natural Emotions in Virtual Reality Through Facial Movement Analysis. Proceedings of the 31st ACM Symposium on Virtual Reality Software and Technology (VRST ’25).

[17] Mavadati M., Sanger P., Mahoor M.H. (2016). Extended disfa dataset: Investigating posed and spontaneous facial expressions. Proceedings of the IEEE Conference on Computer Vision and Pattern Recognition Workshops.

[18] Shomoye M., Zhao R. (2024). Automated emotion recognition of students in virtual reality classrooms. Comput. Educ. X Real..

[19] Cohn J.F., Ambadar Z., Ekman P. (2007). Observer-based measurement of facial expression with the Facial Action Coding System. Handb. Emot. Elicitation Assess..

[20] Martinez B., Valstar M.F., Jiang B., Pantic M. (2017). Automatic analysis of facial actions: A survey. IEEE Trans. Affect. Comput..

